# Uncovering a Phenomenon of Active Hormone Transcriptional Regulation during Early Somatic Embryogenesis in *Medicago sativa*

**DOI:** 10.3390/ijms23158633

**Published:** 2022-08-03

**Authors:** Jianbo Yuan, Yuehui Chao, Liebao Han

**Affiliations:** School of Grassland Science, Beijing Forestry University, Beijing 100083, China; yuanjingan113@163.com

**Keywords:** somatic embryogenesis, *M. sativa*, embryonic induction, embryonic, maturation, DEGs

## Abstract

Somatic embryogenesis (SE) is a developmental process in which somatic cells undergo dedifferentiation to become plant stem cells, and redifferentiation to become a whole embryo. SE is a prerequisite for molecular breeding and is an excellent platform to study cell development in the majority of plant species. However, the molecular mechanism involved in *M. sativa* somatic embryonic induction, embryonic and maturation is unclear. This study was designed to examine the differentially expressed genes (DEGs) and miRNA roles during somatic embryonic induction, embryonic and maturation. The cut cotyledon (ICE), non-embryogenic callus (NEC), embryogenic callus (EC) and cotyledon embryo (CE) were selected for transcriptome and small RNA sequencing. The results showed that 17,251 DEGs, and 177 known and 110 novel miRNAs families were involved in embryonic induction (ICE to NEC), embryonic (NEC to EC), and maturation (EC to CE). Expression patterns and functional classification analysis showed several novel genes and miRNAs involved in SE. Moreover, embryonic induction is an active process of molecular regulation, and hormonal signal transduction related to pathways involved in the whole SE. Finally, a miRNA–target interaction network was proposed during *M. sativa* SE. This study provides novel perspectives to comprehend the molecular mechanisms in *M. sativa* SE.

## 1. Introduction

The process of somatic embryogenesis (SE) consists mainly of dedifferentiation, in which differentiated cells reverse their developmental program during in vitro culture and again during whole-plant development [1,2]. Most plant cells have developmental plasticity, which plays an important role in their reprogramming. The stem cell condition affects the developmental plasticity of plant cells because stem cells are capable of renewing themselves and converting into new somatic embryos that can development into organs or tissues [3]. Chromatin structure is continuously reconstructed throughout plant development, and previous studies showed that chromatin structure plays a crucial role in the pluripotency of plant stem cells [4,5]. In addition, chromatin structure plays important roles in the process of early SE. It is essential, through despiralization of the super-coiled chromatin structure, for the dedifferentiation of somatic cells to produce embryos and induce callus before embryogenesis [4]. On the whole, SE is a powerful tool for research into the processes of plant development and plant stem cell culture conditions [6]. Further investigation of SE will provide opportunities for improving large-scale production of mature somatic embryos and to promote the production of artificial seeds.

Plant growth regulators (PGRs), such as auxin and cytokinins (CKs), are important trigger factors for the culture of plant stem cells for SE [6,7]. For example, 2,4-dichlorophenoxyacetic acid (2,4-D) is an active factor for dedifferentiation in vitro culture, which serves as a “stressor” for the explant [8]. Induction of SE in soybean and potato by 2,4-D is related to increased oxidative stress and expression of defense genes [9,10]. The external PGR supply changes the internal auxin concentration of the explant, which contributes to callus induction [4,9,11].

The process of SE involves various signaling pathways and differential gene expression. Transcription factors (TFs) are also important factors in SE induction. For instance, in Arabidopsis, a marked upregulation of TFs is associated with the embryo induction stage [12]. TFs encoded by genes such as *BABY BOOM* (*BBM*) [13], *PGA6* [14] and *LEAFY COTYLEDON* (*LEC*) [15] also regulate the totipotency of the plant cell, which is critical for SE. *BBM*, which belongs to the AP2/ERF family, is expressed in immature pollen grains of Brassica napus and is expressed preferentially in developing embryos [13]. The *PGA6* gene encodes a homeodomain protein and plays a key role during SE by promoting the vegetative-to-embryogenic transition and maintaining the activity of embryonic stem cells [14]. The *LEC1*, *LEC2* and *LEC3* genes are essential for SE induction in Arabidopsis [15].

Small RNAs guide regulatory processes at the DNA or RNA level in plants. Many small RNAs mediate transcriptional silencing of genes to regulate plant development, whereas other small RNAs mediate post-transcriptional silencing of genes to regulate embryonic development [16]. There are few reports of small RNAs involved in SE, but microRNAs (miRNAs) and other small noncoding RNAs regulate gene expression epigenetically, which plays a crucial role in SE [17]. miR159 regulates *LaMYB33* during the process of SE in *Larix kaempferi* (Lamb) Carr [18]. Several miRNAs, including miR397 and miR156, show positive patterns of expression during the process of dedifferentiation to redifferentiation in rice [19]. *DCL1* regulates miRNA biogenesis during early SE development in Arabidopsis, and the single mutant of dcl1 causes a loss in miRNA156 expression, which results from derepression of *SPL10* and *SPL11* genes [20].

Genome-wide profiling has made it possible to understand molecular regulatory mechanisms of SE. Recently, high-throughput sequencing technology has allowed multiple advances in genome-wide screening of quantitative gene expression in plants [21]. Gene chip technology has been used to determine mRNA abundance and to identify characteristic changes during dedifferentiation in soybeans [22]. The results of the studies mentioned imply that new cells of dedifferentiation generation that develop organized structures may rely on gene regulation to balance cell proliferation and cell death [9]. Proteomic analysis suggests the involvement of mechanisms in the transition from morphologically mature to physiologically mature somatic embryos during the partial desiccation treatment process in *Picea asperata* [23]. These studies help to identify the molecular regulatory mechanisms that are active during SE.

*M. sativa* is a tetraploid perennial species that is the most important cultivated forage crop due to its high regeneration capacity in vitro. Thus, *M. sativa* has been used in molecular studies and breeding. The first reported *M. sativa* regeneration was accomplished via SE [24]. In the past, the study of somatic embryo formation in *M. sativa* focused mainly on the morphological and physiological levels; the molecular mechanisms related to SE in *M. sativa* remain unclear. For example, in proteomic analysis of SE in two varieties of M. truncatula, 6-benzylaminopurine (BAP) and 1-naphthaleneacetic were added to the explant culture medium. The results suggested that more than 60% of differentially expressed protein spots exhibited different patterns of gene expression between the two varieties during 8 weeks of culture [25]. This study aimed to identify molecular mechanisms during somatic embryonic induction, embryonic and maturation in *M. sativa*.

In this study, we used Illumina high-throughput sequencing technology to analyze DEGs and miRNAs expression at the ICE, NEC, EC and CE phases in *M. sativa* SE. A total of 17,251 DEGs 177 known and 110 novel miRNAs families were obtained. Among these, several novel DEGs and miRNAs were detected during somatic embryonic induction, embryonic and maturation. For example, novel_247 targets the *LTP8* gene, which may play an important role in maturation. In addition, the results suggest a potential miRNA–target gene interaction network involved in *M. sativa* SE, which further complements the molecular mechanisms in SE.

## 2. Results

### 2.1. Morphological Comparison of SE at Different Developmental Stages

**Figure 1:**
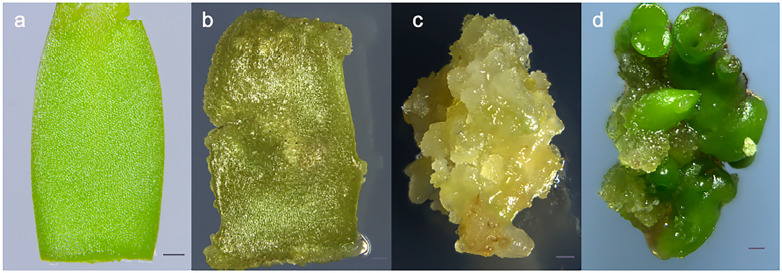
Somatic embryogenesis in alfalfa at four developmental phases used for RNA-Seq analysis; (**a**) Cotyledon cutting; (**b**) Non-embryogenic callus; (**c**) Embryogenic callus; (**d**) Cotyledon embryo. Bar = 1 mm.

**Figure 2:**
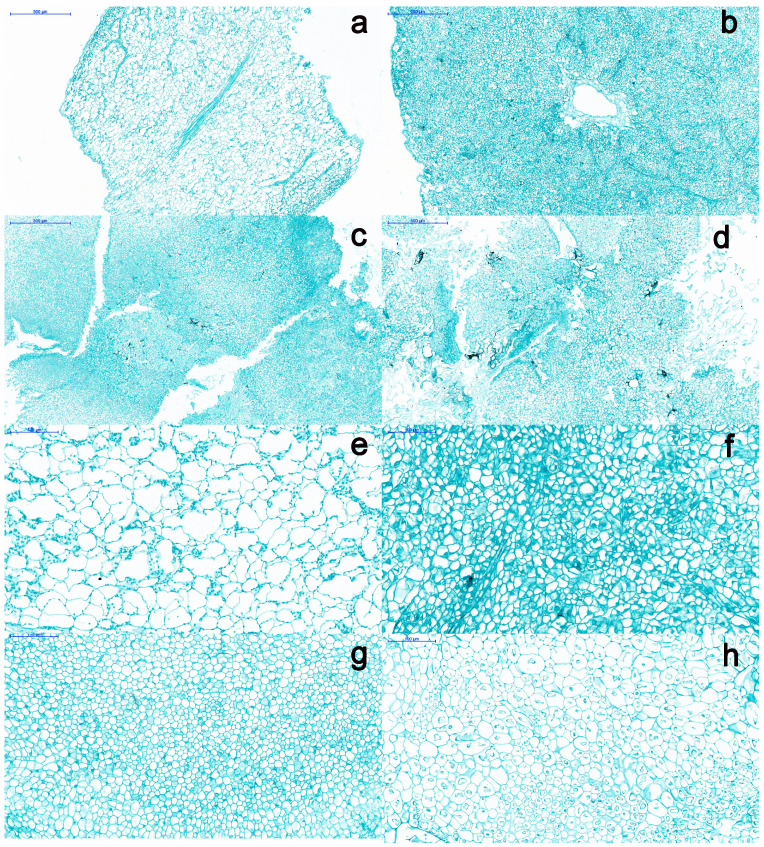
Morphology of somatic embryogenesis callus. (**a**) cut cotyledon; (**b**) non-embryogenic callus; (**c**) embryogenic callus; (**d**) cotyledon embryo; (**e**) Cut cotyledon; (**f**) non-embryogenic callus; (**g**) embryogenic callus, bar = 100 μm; (**h**) cotyledon embryo.

SE involves three phases: embryonic induction, embryonic and maturation [26]. To understand the detailed phases of SE, 14 developmental stages were observed using a light microscope (Figure S1). We selected four stages (ICE, NEC, EC and CE) for RNA sequencing (RNA-seq), as the morphological changes observed in these four stages were the most notable. The stages from ICE to NEC represent the embryonic induction process (Figure 1a,b and Figure 2a,b,e,f), NEC to EC represents the embryonic stage (Figure 1b,c and Figure 2b,c,f,g), and EC to CE represents the maturation process (Figure 1c,d and Figure 2c,d,g,h). The cell size during the ICE was larger than during the NEC, EC and CE, whereas the number of cells was larger in the EC than ICE, NEC and CE. The cell shape was more regular in the EC than the ICE, NEC and CE. From ICE through EC, the cell number increased gradually, while the cell size decreased. However, from the EC to the CE phase, the cell size increased gradually, while the cell number decreased gradually. These findings suggest that the changes in tissue morphology differed significantly among the various development stages.

### 2.2. Transcriptome Sequencing and Assembly

**Figure 3:**
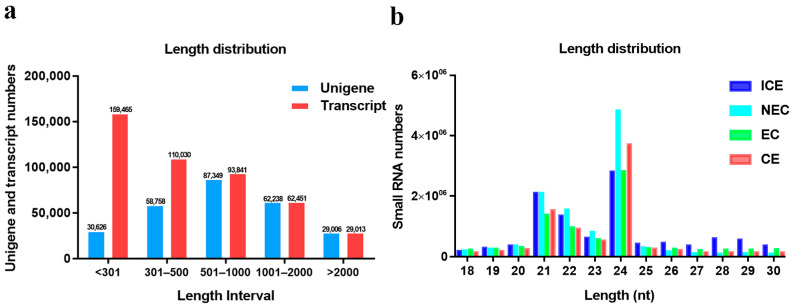
Unigene and small RNA length distributions. (**a**) Unigenes and transcriots; (**b**) Small RNAs.

ICE, NEC, EC and CE cells were used as sources for RNA-seq. All data were generated using three biological replicates. The RNA-seq generated an average of 8.15 GB of data for the ICE, NEC, EC and CE databases (Table 1). The false discovery rate was less than or equal to 0.02%. All databases produced a total of 668,180,574 raw reads, including 97.60% Q20 bases with a 41.60% GC content. After 97.53% of the raw reads were selected for filtration, 207, 276, 776 clean reads were selected for further analysis using Trinity. All clean reads were assembled into 267, 977 unigenes. The mean length of the genes was 986 bp, and the maximum length was 16,765 bp. The N50 fragment length was 1392 bp, and the N90 fragment length was 466 bp. The size distributions of the unigenes and transcripts are shown in Figure 3a. Unigenes of 501–1000 bp were the most common in all sample data, accounting for 32.6% of the data. Transcripts of 301 bp or less were the most frequent, accounting for 35.85% of all data.

### 2.3. Gene Annotation and Functional Classification in M. sativa

After matching the sequences with the KOG/COG database, a total of 43,572 unigenes were classified into 26 categories (Figure S2). The majority of unigenes (5207) were predicted to be associated with post-translational modification, protein turnover and chaperones (11.95%), followed by general functions (11.90%), translation, ribosomal structure and biogenesis (8.18%), signal transduction mechanisms (7.73%) and RNA processing and modification (6.85%). Five unigenes (0.01%) had unknown functions.

A total of 101,969 unigenes were selected for GO classification using Blast2GO v2.5 (BioBam, Valencia, Spain), which classified into biological process, cellular component and molecular function groups (Table S1). The highest category was nucleoside binding (14.03%) of molecular function, followed by intracellular membrane-bounded organelle (12.84%), small molecule metabolic process (10.77%), cytoplasm (8.78%) and cell communication (7.55%). In the cellular component, many of unigenes conducted various functions, which contained nuclear chromatin (0.01%), cell wall (0.04%), plasma membrane (1.07%) and transporter complex (0.35%). In the distribution of molecular function, the nucleoside binding (14.03%), substrate-specific transmembrane transporter activity (4.29%) and hydrolase activity, acting on ester bonds (3.65%) were mainly representation. Interestingly, 83 unigenes were predicted to be involved in embryonic development and 237 in post-embryonic development. The unigenes associated with embryogenesis are summarized in Table S2, which includes 39 GO functional terms. There were embryo development (83), reproductive structure development (34), regulation of cell differentiation (49), post-embryonic morphogenesis (201) and root morphogenesis (3), these GO terms were important developmental processes during SE.

All unigenes were analyzed using KEGG classification to identify the biological functions in *M. sativa*. After mapping against the KEGG database, 47,092 unigenes were classified into four main categories and 128 pathways (Table S3). The largest category of unigenes was metabolism, accounting for 62.7%, followed by genetic information processing (26.89%), cellular processes (6.41%) and environmental information processing (4%). Interestingly, carbon metabolism was the largest representative in the metabolism category, accounting for 4.66% of unigenes. This finding was consistent with that described in *Lilium* SE [27], suggesting that carbon metabolism is active in *M. sativa*. In addition, zeatin biosynthesis pathway (182) components of metabolism and plant hormone signal transduction genes involved in environmental information processing (1046) were predicted to be active in *M. sativa* SE, suggesting that these pathways play important roles in SE.

### 2.4. Identification and Analysis of DEGs in SE

**Figure 4:**
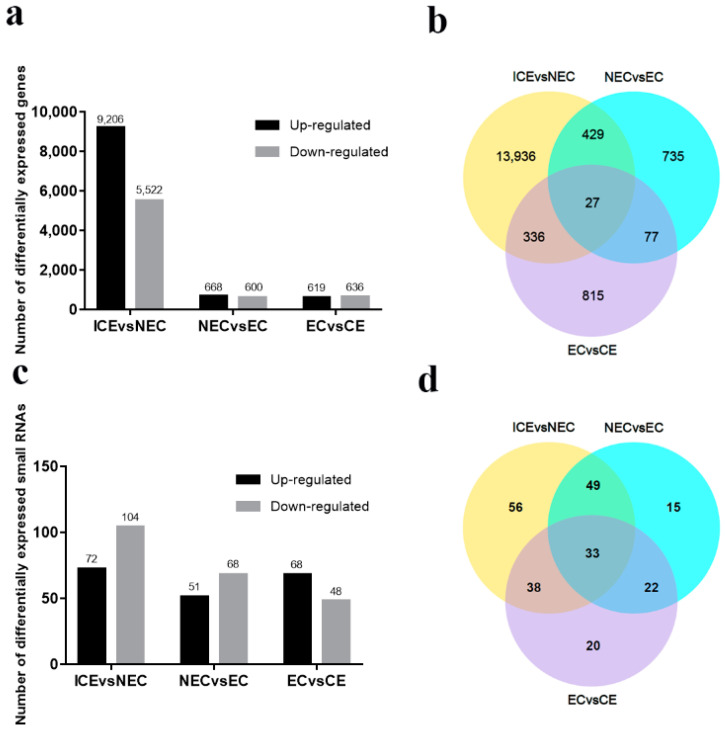
Histograms and Venn diagrams of DEGs for three phrases in SE: embryonic induction (ICE vs. NEC), embryonic induction (NEC vs. EC), and maturation (EC vs. CE). (**a**) The numbers of DEGs up- or down-regulated during SE; (**b**) Venn diagram showing similarly or differently regulated genes over the three phreases; (**c**) The numbers of small RNAs up- or down-regulated during SE; (**d**) Venn diagram showing similarly or differentially regulated small RNAs during SE.

After four unigene libraries were compared with each other to identify DEGs (*p*-value < 0.05 and |log2 FC| ≥ 1), three DEG groups were then produced (ICE vs. NEC, NEC vs. EC, EC vs. CE) that included 17,251 DEGs (Table S4). A comparative analysis of the 17,251 DEGs at different SE phases is shown in Figure 4a. The number of DEGs was significantly higher in the ICE compared to the NEC, and higher in the NEC compared to the EC, and in the EC compared to the CE. In addition, 9206 genes were upregulated and 5522 genes were downregulated in the ICE compared to the NEC. Only 668 genes were upregulated and 600 genes were down-regulated in the NEC compared to the EC, and 619 genes were upregulated and 636 genes were downregulated in the EC compared to the CE. These results indicate that the early SE is active during the complicated development process.

All DEGs were selected for Venn diagram analysis (Figure 4b). In total, 27 genes were expressed in all the phases of SE, 429 genes were expressed in the ICE to the NEC and the NEC to the EC two development phrases; 77 genes were expressed in the ICE to NEC, and the EC to the CE two development groups; 336 genes were expressed in the ICE to NEC and NEC to EC two development phrases. There were few DEG involved in multiple SE stages.

### 2.5. Some DEGs Involved in Embryonic Induction, Embryonic and Maturation

To further investigate the function of DEGs, we identified 61 DEGs in 18 families involved in SE (Table S5), including *BABY BOOM* (*BBM*), *WUSCHEL related homeobox* (*WOX*), *PKL* (*PICKLE*) and somatic embryogenesis receptor-like kinase (*SERK*). Most of the DEGs (80.32%) were involved in embryonic induction, followed by embryonic (14.75%) and maturation (4.91%). In addition, *LATERAL ORGAN BOUNDARIES DOMAIN* (*LBD*), *argonaute* (*AGO*), *glycogen debranching enzyme* (*AGL*) and authentic response regulator (*ARR*) were observed in embryonic induction and embryonic, and *WOX* and *Pyoluteorin* (*PLT*) were involved in embryonic induction and maturation. Interestingly, no single gene family was observed throughout SE, indicating that stage-specific studies are necessary in the future. Among all DEGs, 14 were upregulated and 35 downregulated in embryonic induction, particularly *LBD41*, *LBD16*, *flavin monooxygenase5* (*YUC5*) and *YUC9*, with |log2 FC| values > 9. 5 genes were upregulated and 3 genes were downregulated in embryonic. Such as *PKL* and *LBD4* were upregulated more than 3-fold. *WOX6*, *PLT1* and *PLT2* was upregulated more than 3-folds in maturation.

### 2.6. Expression of Plant Hormone Signal Transduction Genes in SE

**Figure 5:**
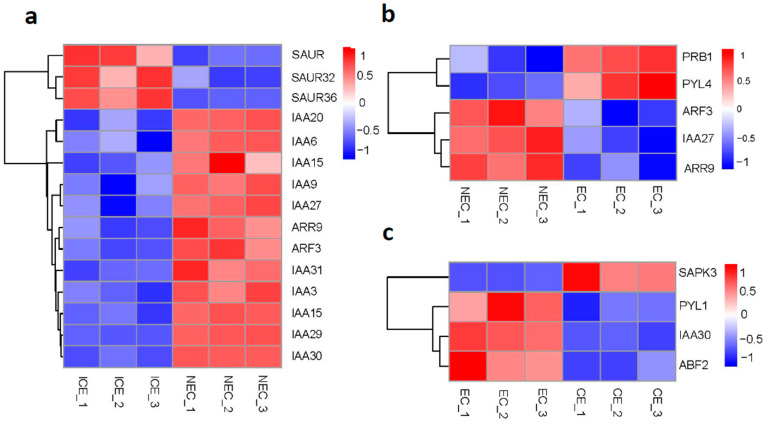
Plant hormone signal transduction genes in SE. (**a**) Expression of gene in ICE to NEC; (**b**) Expression of gene in NEC to EC; (**c**) Expression of gene in EC to CE. ∣log2FC∣ > 0.5. ICE_1, ICE_2 and ICE_3 represent three biological replicates.

**Figure 6:**
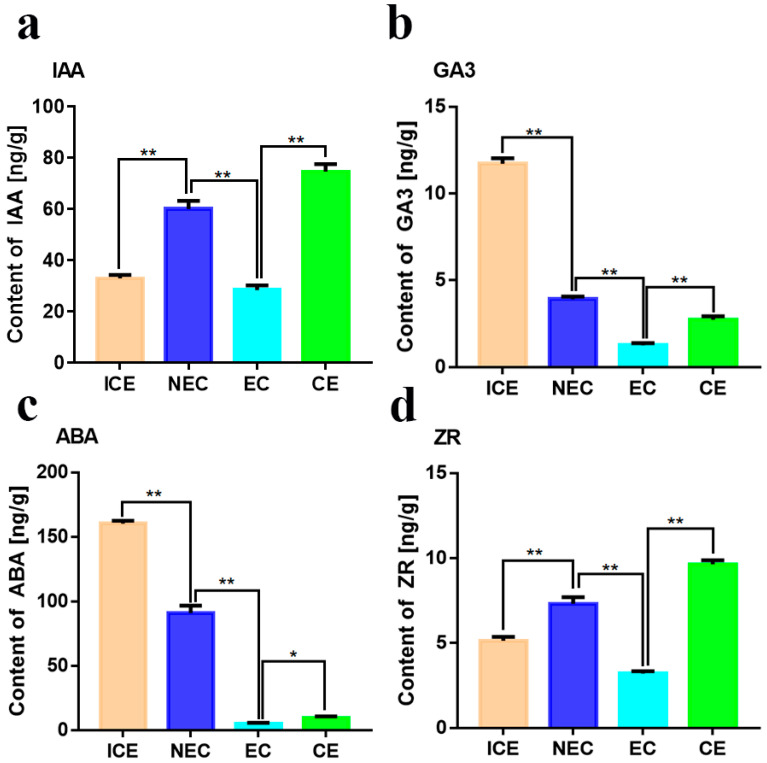
Hormone changes during different phases of SE. (**a**) Auxin changes at cut cotyledon (ICE). non-embyogenic callus (NEC), embyogenic callus (EC), and cotyledon embryo (CE) phases; (**b**) Gibberellin changes at ICE, NEC, EC and CE phases; (**c**) Abscisic acid changes At ICE, NEC, EC and CE; (**d**) Cytokinin changes at ICE, NEC, EC and CE. Indirect competitive enzyme-linked immunoassay (icELISA) for hormone detection. * *p* < 0.05, ** *p* < 0.01 (Student’ s *t*-test).

We detected ~118 DEGs involved in plant hormone signal transduction pathways (Table S6), including IAA, zeatin, ethylene (ET), ABA and GA pathways; 106 DEGs were involved in the transition from embryonic induction (Table S6), 8 in maturation, and 5 in embryonic (Table S6). Several DEGs exhibited higher expression levels during embryonic induction. *IAA*, *ARR*, and *Auxin Response Factor* (*ARF*) family genes were upregulated more than 2-fold (|log2 FC| > 2). However, Auxin-induced protein (SAUR), *SAUR32* and *SAUR36* was downregulated (Figure 5a). KEGG analysis indicated that *IAA*, *ARF* and *SAUR* are involved in auxin pathways during embryonic induction. Endogenous auxin expression increased nearly 2-fold from ICE to NEC (Figure 6a). Elhiti et al. found that expression of *LEC* gene directly induce AGAMOUS expression during early embryogenesis, which in turn upregulates *GA_2OX_* and decreases GA synthesis [26]. In our data, which are *GA_2OX_* homologs as DEGs. During the embryonic induction, LEC2 gene expression was upregulated more than 5-fold, and *GA_2OX1_* was downregulated more than 2-fold. However, *GA_2OX2_* was downregulated more than 3-fold. The expression of these genes did not change significantly during other phases. GA3 expression decreased sharply at four sampling sites (Figure 6b). Li et al. found that several MYBs are positive regulators of ABA responses [28]. We also detected genes homologous to MYB among the DEGs, including *MYB4* and *MYB48*. In maturation, *MYB4* and *MYB48* were downregulated more than 2.03-fold, with no significant changes during the other phases. We detected changes in ABA among the phases (Figure 6c). From ICE to NEC and NEC to EC, the level of ABA decreased sharply. However, from EC to CE, the ABA content increased slightly. We also detected changes in ZR levels among the phases (Figure 6d). From ICE to NEC, the ZR content was slightly upregulated, and from NEC to EC, it was decreased by nearly half. However, from EC to CE, the ZR content more than doubled. In addition, *Pathogenesis-related protein 1* (*PRB1*) and *Abscisic acid receptor* (*PYL4*) were upregulated, and *IAA27*, *ARF3* and *ARR9* were downregulated in embryonic (Figure 5b). *Serine/threonine-protein kinase* (*SAPK3*) was upregulated, and *PYL1*, *IAA30* and *ABA response element-binding factor* (*ABF2*) were downregulated in maturation (Figure 5c).

### 2.7. Annotation of Small RNA-Seq Data

To investigate SE in *M. sativa*, four small RNA libraries (ICE, NEC, EC and CE) were sequenced. A total of 51,465,356 raw reads were obtained from each library. After filtering, 46,453,585 clean reads were obtained from four libraries (Table 2). The most common small RNAs were 21–24 nucleotides (nt) in length (Figure 3b), with the majority being 24 nt. All unique sequences were annotated and mapped in the Rfam database using BLAST. A total of 19,765,615 small RNAs were annotated in four libraries. ICE was the most abundant, accounting for 30.19% (Table 3). The annotated small RNAs included known miRNAs, novel miRNAs, ribosomal RNAs (rRNAs), tRNAs, snRNAs, small nucleolar RNAs (snoRNAs) and trans-acting small interfering RNAs (TASs) in ICE. The known miRNAs were the most abundant (12.04%), followed by novel rRNAs (7.24%), novel miRNAs (2.23%), TASs (0.32%), snoRNAs (0.16%), snRNAs (0.07%) and tRNAs (0.00%). However, 77.58% of the other small RNAs involved in ICE are unknown. These results indicated that small RNAs are more active in embryonic induction than in other development stages.

### 2.8. Identification and Expression Analysis of Known miRNAs in M. sativa

**Figure 7:**
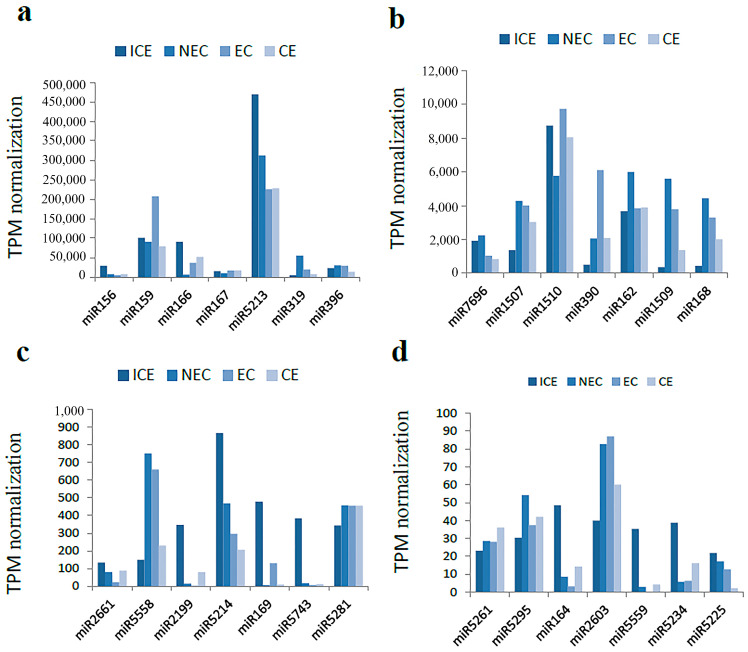
Transcripts reads per million reads known miRNAs in *Medicago sativa*. (**a**) miRNAs families with TPM value over 10,000; (**b**) miRNA families TPM value between 1000 and 10,000; (**c**) miRNA families TPM value between 100 and 1000; (**d**) miRNA families TPM value between 0 and 100.

After annotating the small RNAs, ~308 known miRNAs were identified from four libraries. Table S7 shows the number of known miRNAs. All identified known miRNAs belong to 177 miRNA families. Several miRNA families contained more than one member, such as the mi156 (11), miR166 (5), miR169 (7) and miR171 (8) families. Approximately 45 families contained only one member, such as the miR5218, miR5237, miR2625 and miR2605 families. Small RNA-seq data revealed 124 known families with significantly different expression levels ranging from 0 to 1,000,000 transcripts per kilobase million (TPM) among all libraries (Table S8). These 124 families were classified into four groups based on maximum expression levels. The first group contained 13 families, including miR5213, miR159, miR166 and miR167, which expressed more than 10,000 TPM and were detected in at least one sample. Among these 13 families, miR5213 showed the highest expression, exceeding 200,000 TPM in each sample (Figure 7a). The second group contained 11 known families that ranged in expression from 1000 to 10,000 TPM, with miR1510 showing the highest expression of more than 5000 TPM in all samples (Figure 7b). The third group contained 21 families that ranged in expression from 100 to 1000 TPM, with miR5214 exhibiting the highest expression level in this group (Figure 7c). The fourth group contained the remaining 79 families, which ranged in expression from 0 to 100 TPM. As shown in Figure 7d, miR2603 showed the highest level of expression.

### 2.9. Identification of Potential Novel miRNAs in M. sativa

The miREvo and miRdeep2 software (Berlin, Germany) were used to predict several potential miRNAs due to their unique hairpin structure. This mature sequence is a common trait in potential novel miRNAs in *M. sativa*, and these novel miRNA candidates might be regarded as a new miRNA family if they originated from different loci. We identified 110 novel families distributed in four samples (Table S9). Interestingly, 10 families were involved throughout SE (novel_322, novel_263, novel_249, novel_70, novel_67, novel_186, novel_94, novel_46, novel_231 and novel_287), which accounting for 9.09%. We detected 21 families that were expressed exclusively during embryonic induction, 12 during maturation and 9 during embryonic. The length of the novel family members expressed during the maturation phase ranged from 19 to 24 nt. Gene expression profiles revealed that 28 families were upregulated and 46 families downregulated in embryonic induction, and 27 families were upregulated and 30 families downregulated in embryonic formation. More families were downregulated than upregulated during the embryonic induction and embryonic formation stages. However, more families were upregulated (32) than downregulated (24) during the maturation stage. These results suggest that several new novel miRNAs play roles in maturation in a manner that differs from those involved in embryonic induction and embryonic.

### 2.10. Expression of Known and Novel miRNAs during SE in M. sativa

The numbers of miRNAs involved in the three developmental processes are shown in Figure 4c. The miRNA changes from the ICE to the NEC phase were significantly higher than in the NEC to the EC phase and the EC to the CE phase. From the EC to CE stage, more miRNAs were upregulated than downregulated. Venn diagram analysis of 418 miRNAs showed different expression levels among the three developmental processes (Figure 4d). A total of 33 miRNAs were expressed throughout SE; 49 miRNAs overlapped between the ICE to the NEC phase and the NEC to the EC phase, 22 miRNAs overlapped between the ICE to the NEC phase and the EC to the CE phase, and 38 miRNAs overlapped between the ICE to the NEC phase and the EC to the CE phase.

**Figure 8:**
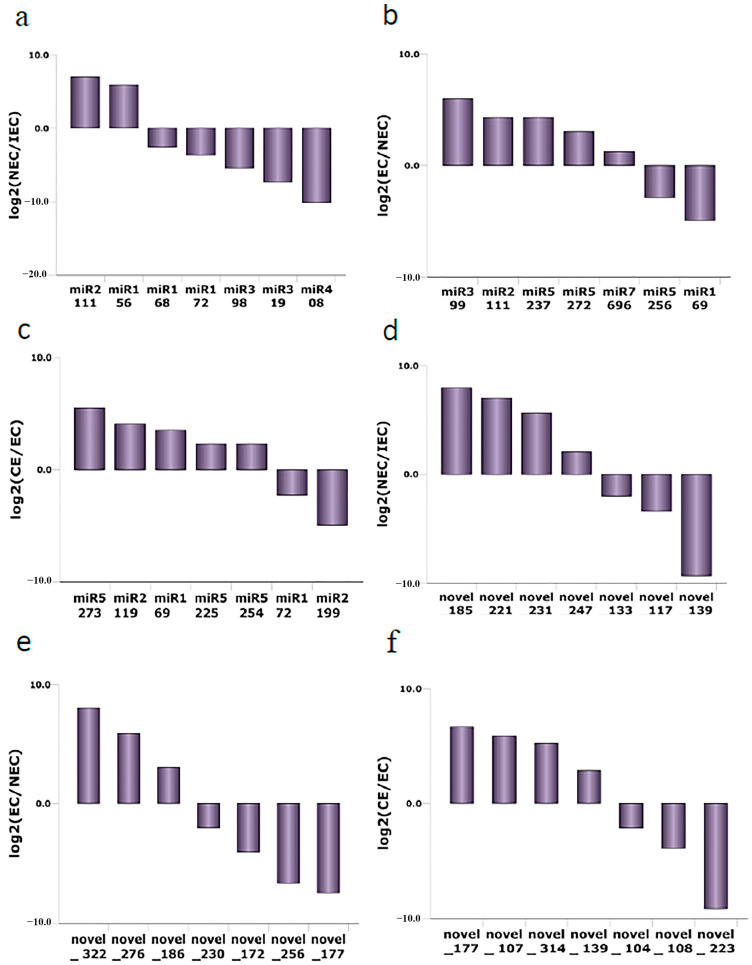
Differentially expressed known and novel miRNAs in *M. sativa* SE. (**a**–**c**) comparison of diferentially known miRNA families among ICE to NEC, NEC to EC and EC to CE; (**d**–**f**) comparison of diferentially novel miRNA families among ICE to NEC, NEC to EC and EC to CE. ∣log2FC∣ ≥ 2.

As mentioned previously, SE in *M. sativa* was classified into three major stages: embryonic induction (ICE to NEC), embryonic (NEC to EC) and maturation (EC to CE). The differentially expressed miRNA families were defined as having an |log2 FC| value ≥ 2. A total of 39 known miRNA families exhibited differential expression during embryonic induction. Among these families, seven were selected for further comparison analysis in embryonic induction; five families showed downregulated expression of varying degrees, and two families were upregulated, including miR156 and miR2111 (Figure 8a). A total of 18 known miRNA families showed differential expression during the embryonic phase. We selected seven of these families for analysis (Figure 8b), of which five were upregulated and two were downregulated (miR5256 and miR169). A total of 17 known miRNA families were differentially expressed during maturation. Five families were upregulated and two downregulated (Figure 8c). In general, these results indicate that each developmental stage relies on major clusters of known miRNAs to regulate SE in *M. sativa*.

To identify the major miRNA clusters involved in the various stages of SE, we analyzed the differential expression of 110 novel miRNA families. A total of 51 families showed differential expression in embryonic induction, 33 in embryonic formation and 32 in maturation. We selected seven families for analysis in each stage. There was no significant difference in the number of miRNAs that were up- or downregulated (Figure 8d–f). Interestingly, the novel miRNA families showed differential expression in each stage, a phenomenon similar to that observed with known miRNAs. These results indicated that several major novel miRNA clusters showed stage-specific expression.

### 2.11. Analysis of Target Genes of miRNAs

To investigate the miRNA-mediated pathways during different stages of SE, we analyzed 17,253 unigenes with annotations from transcriptome databases and used the psRNATarget website to predict target genes. We identified 5785 potential target genes potentially related to 408 miRNAs (Table S11). More than 92.89% of miRNAs had multiple target unigenes, and only 29 miRNAs (7.11%) had a single unigene or no unigene from all miRNA–target gene pairs. Among the miRNA–target gene pairs, most 43.14% (176) were detected during embryonic induction (Table S10); 29.16% (119) were detected in the embryonic stage and 27.7% (113) in the maturation stage. These results suggest that the embryonic induction stage was the most active period. The prediction of miRNA–target pairs suggests that miRNAs can mediate multiple pathways during the various phases of SE in *M. sativa*. Most target genes exhibited significant differences in expression, revealing a complex biological regulation process in SE. These target genes included hormone-related genes, such as *ARF*, *LBD*, and *ARR2*, TFs and certain kinases. KEGG analysis showed the involvement of these target genes in plant hormone signal transduction pathways. As *ARF* is involved in tryptophan metabolism, it may function to promote cell growth in SE. This study showed the involvement of four members of the ARF family in *M. sativa* SE (Table S11). *ARF17*, *ARF18* and *ARF10* were targeted by miR160, whereas ARF8 was targeted by miR156, miR167 and novel_272 in embryonic induction (Table S11). *LBD11* was targeted by miR7696 in embryonic. miR2604 targeted *ARR2*, which is involved in the zeatin biosynthesis pathway that promote cell division in SE. In addition, we detected several TFs related to embryonic development, such as *SHORT-ROOT* (*SHR*) and *Polyol transporter 5* (*PLT5*). *SHR* was targeted by miR156 in embryonic induction, and *PLT5* was targeted by *novel_299* in embryonic. Several kinase genes were targeted in SE; SERK5, mitogen-activated protein kinase 8 (Mapk8) and cyclin-dependent kinase C-1 (CDKC-1) were targeted by miR5561, miR5559 and miR7701, respectively. 

To further investigate the function of miRNAs in SE, 5785 target genes were selected for GO analysis. We identified 1350 GO terms involved in embryonic induction, 416 in maturation and 266 in embryonic (Table S12). The biological (GO: 0008150), metabolic (GO: 0008152), cellular (GO: 0009987), organic substance metabolic (GO: 0071704) and primary metabolic (GO: 0044238) processes were the main biological process categories enriched among the target genes. Among the cellular categories, cellular component (GO: 0005575), cell (GO: 0005623) and cell part (GO: 0044464) accounted for the highest proportion of target genes. Molecular function (GO: 0003674), binding (GO: 0005488) and catalytic activity (GO: 0003824) were the enriched molecular function categories among the target genes. As shown in Table S12, several miRNAs with target genes were involved in biological processes related to SE in *M. sativa*. For example, novel_249 play a role in post-embryonic development, plant epidermal development, and regulation of multicellular organismal development; novel_211 regulate cell growth and cell differentiation in SE; and miR2673 play an important role in cell development, cell–cell signaling, and cell activation. Interestingly, 22 miRNA families with 44 target genes were involved in cellular developmental processes, representing the most active biological processes in SE. In addition, several miRNAs were involved in programmed cell death, such as miR5207, miR2630 and miR319.

**Figure 9:**
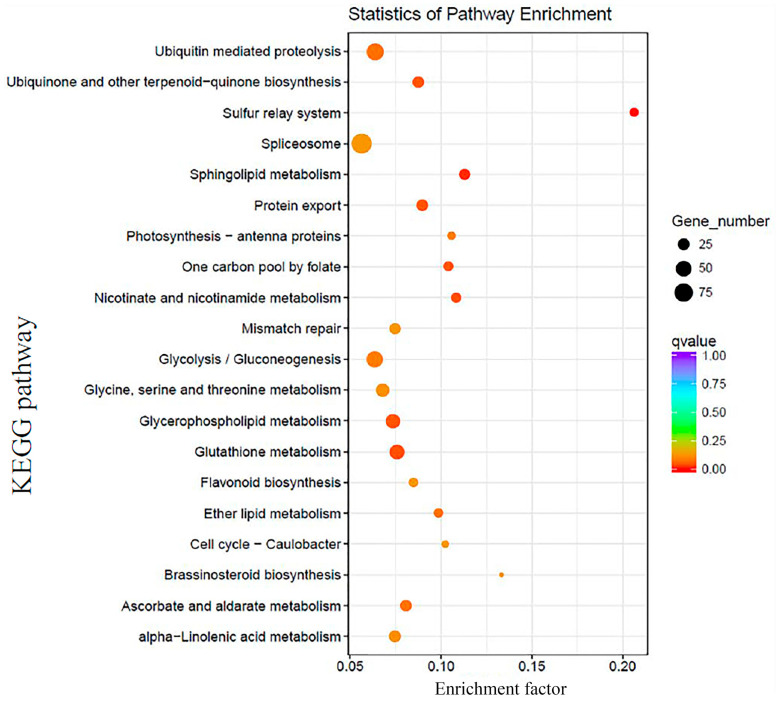
KEGG enrichment target genes in *M. sativa* SE.

A total of 5785 target genes showed KEGG enrichment using the KOBAS, revealing the involvement of 112 pathways and 1953 target genes in SE (Table S13). The spliceosome pathway showed the highest enrichment (91 target genes), followed by protein processing in the endoplasmic reticulum (62) and ubiquitin-mediated proteolysis (61) (Figure 9). Moreover, several other pathways may play key roles in M. sativa SE, such as plant hormone signal transduction, amino sugar and nucleotide sugar metabolism, and RNA degradation (Table S13).

### 2.12. Validation of Various Expression Patterns of DEGs and miRNAs

**Figure 10:**
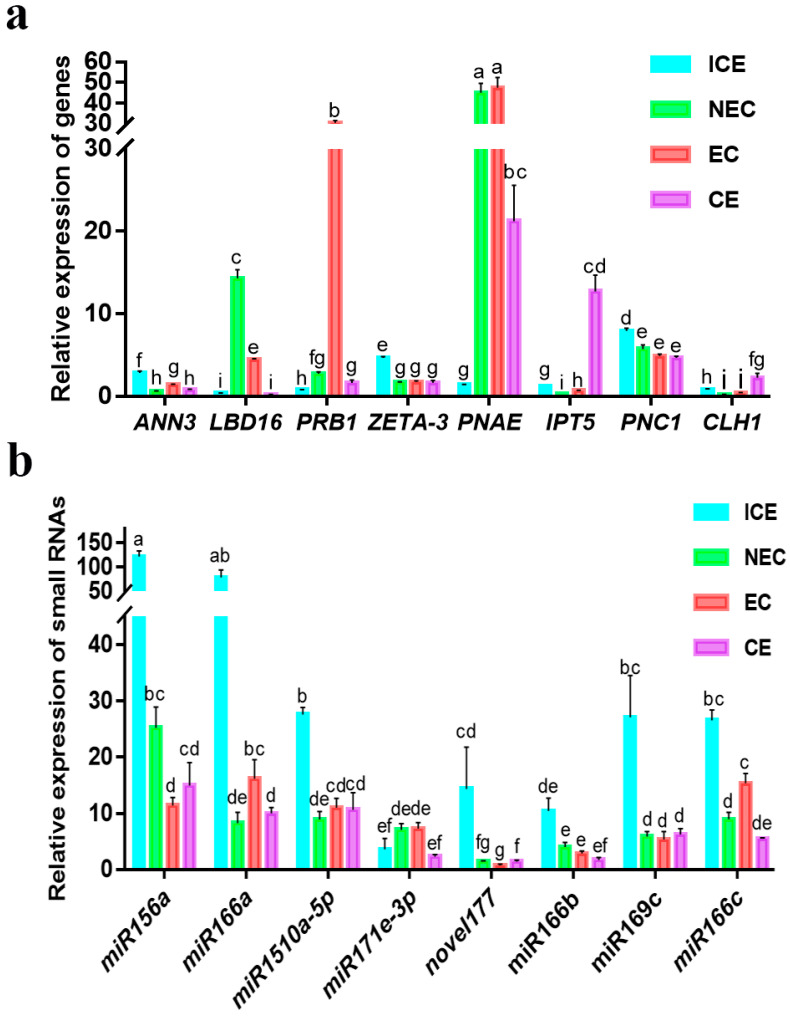
Relative expression of genes and miRNAs during cut cotyledon (ICE), non-embyogenic callus (NEC), embryogenic callus (EC), cotyledon embryo (CE) phases. (**a**) The relative expression of DEGs; (**b**) The relative expression of miRNAs. genes and miRNAs calculated by the equation = 2^−ΔΔCt^, The 18sRNA is housekeeping gene for gene expression detection, and the U6 gene is housekeeping gene for miRNA expression detection. The different letters represent significant differences (*p* < 0.05, (ANOVA)).

To validate the expression patterns of genes and miRNAs during ICE, NEC, EC and CE, we determined the expression levels of several genes and miRNAs by qRT-PCR and transcriptome analysis. qRT-PCR analysis revealed that *PNC1* expression was downregulated in SE (Figure 10a); likewise, transcriptome analysis showed that *Cationic peroxidase 1* (*PNC1*) was downregulated more than 4-fold throughout SE. Our results showed that *PNC1* was targeted by novel_244, and novel_244 was downregulated slightly from ICE to NEC. The *IPT5* expression level did not differ significantly from ICE to EC. However, *IPT5* gene expression was significantly upregulated from EC to CE (Figure 10a). Similarly, the transcriptome results showed that *IPT5* was upregulated more than 5-fold from EC to CE. However, no obvious changes in *IPT5* levels were observed in the other stages. miR156 families play crucial roles during SE. From ICE to NEC, the miR156a expression level was downregulated more than 3-fold (Figure 10b), as revealed by small RNA-seq data. *SPL6* was targeted by miR156a, which was upregulated from ICE to NEC. No obvious changes in miR156a levels were observed in the other stages. miR166a expression was downregulated from ICE to NEC and EC to CE and upregulated from NEC to EC. Small RNA-seq and qRT-PCR analysis of miR156a showed consistent results. *2-succinylbenzoate--CoA ligase* (*AAE*) was targeted by miR166a. *AAE* was upregulated from ICE to NEC but showed no obvious changes in the other stages.

## 3. Discussion

SE, a complex developmental process involved in completing plant regeneration of somatic cells, was first described in the carrot [29,30]. Previous studies identified single or multiple genes involved in SE using mRNA differential display [31]. However, the more recent technology of RNA-seq has clear advantages, as it can be used to generate millions of clear reads for transcriptome and miRNA analyses. Xu et al. suggested that these analyses can be used to generate a comprehensive view of several gene and miRNA families involved in SE in plants [32]. However, no such studies of DEGs or miRNAs in SE in *M. sativa* have been reported. This study investigated DEGs and miRNA roles during somatic embryonic induction, embryonic and maturation in *M. sativa* SE. Four libraries from four SE stages (ICE, NEC, EC and CE) were constructed, and pairwise comparisons of the data resulted in annotation of 101,969 unigenes and 418 miRNAs and prediction of several target genes of miRNAs. Finally, we proposed a potential miRNA–target interaction network involved in *M. sativa* SE.

### 3.1. The Early Phase of SE Is an Active Process of Molecular Regulation

Although numerous studies have examined the mechanism of SE in the past decade [4,33], the molecular mechanisms underlying somatic embryonic induction, embryonic and maturation in *M. sativa* is unclear. The embryonic induction can be used to study early embryonic development. In the embryonic induction phase, which occurs early in SE, 9206 genes were upregulated and 5522 genes downregulated, with greater numbers of DEGs detected than during the other phases of SE. We identified several genes that play a regulatory role in early somatic embryonic development, including polycomb repressive complex (*PRC1*), *WUS*, *SERK1* and *heat shock protein17* (*HSP17*) (Table S4). *PRC1* and *PRC2* modify chromatin to repress the expression of genes not required for a specific differentiated state [34]. *WUS* is a marker of embryonic cells [26], and several studies have shown that *WUS* is important in totipotent embryogenic stem cells [14,26]. *SERK1* encodes a leucine repeat receptor protein kinase, which promotes early embryogenesis [35]. *HSP17* shows transient accumulation during embryonic maturation and germination in the oak and increases in level during dedifferentiation in SE [36]. In addition, we identified several new genes that were upregulated 10-fold from the embryonic induction phase, including *Phosphoglycolate phosphatase 1B* (*PGLP1B*), *Benzyl alcohol O-benzoyltransferase* (*HSR201*) and *Probable glucuronoxylan glucuronosyltransferase* (*IRX7*) (Table S4). Therefore, we infer that the early phase of SE in *M. sativa* involves an active process of molecular regulation.

### 3.2. Identification of Hormonal Signal Transduction during SE in M. sativa

Plant hormones and PGRs play critical roles during SE. Plant hormones specify the endogenous compounds produced by diverse cells, and PGRs complement synthetic compounds added exogenously [37]. Among all phytohormones, auxin plays important roles in regulating plant development, while IAA has been recognized as the most important auxin [38]. Auxin biosynthesis is regulated by *Aux/IAA*, *TIR1*, *ARF*, *CH3* and *SAUR* [39,40,41,42,43]. Our study suggests that the key genes *Aux/IAA*, *ARF*, *CH3* and *SAUR* are involved in auxin biosynthesis. The expression of *Aux/IAA* and CH3 were significantly upregulated in the embryonic induction and maturation stages. Auxin/IAA (Aux/IAA) proteins are transcriptional regulators of plant responses to auxin during fruit development and leaf morphogenesis. *IAA9* belongs to the Aux/IAA gene family and is downregulated in Arabidopsis mutants [44].

Cytokinins play important roles in promoting cell division [6]. Cytokinin biosynthesis is regulated by *CRE1*, *histidine phosphotransfer proteins* (*AHPs*), *A-ARRs* and *B-ARRs* [45,46,47,48]. *AHPs*, *A-ARRs* and *B-ARRs* are involved in embryonic induction. *A-ARRs* are also involved in embryogenic. The genes encoding *AHP*, *A-ARR* and *B-ARR* exhibited significant downregulation during embryonic induction, whereas *A-ARR* showed upregulation during the embryogenic formation stage. *AHP* is a phosphorelay carrier between cytokinin receptors and nuclear cytokinin responses. AHP mutants function study exhibited that AHP were positive factors in cytokinin signaling [49,50]. *B-ARRs* are mediators of cytokinin signaling, while *A-ARRs* are negative regulators of cytokinin signaling that function via feedback mechanisms to the primary cytokinin signal response [48,51].

GA regulates cell elongation during seedling development [52]. Several TFs are involved in GA biosynthesis during SE in *M. sativa*, including *PHYTOCHROME INTERACTING FACTOR* (*PIF4*), *NONEXPRESSOR OF PRGENES1* (*NPR1*), *ABRE-BINDING FACTORS* (*ABF*), *SALT-RESPONSIVE ERF1* (*ERF1*), *bHLH transcription factors* (*MYC2*) and *Thermal Gravimetric Analysis* (*TGA*). *NPR1*, *ABF* and *ERF1* showed significant upregulation in embryonic induction. In addition, *ABF* showed positive upregulation in maturation. ABF is a key factor involved in the transition of embryogenesis to seed germination, playing an intermediate role in the cross-talk between ABA and GA signaling [53]. *NPR1* regulates the cross-talk between salicylic acid and jasmonic acid signaling pathways [54]. As a role of *NPR1* in GA synthesis has not been reported, further studies are required. *ERF1* is involved in not only the ET signaling pathway but also the ABA and GA biosynthetic pathways [55].

ABA supplementation promotes SE during maturation in *Podocarpus lambertii* [56]. Our study showed that ABA biosynthesis plays important roles in SE in *M. sativa*; the key molecules regulating ABA biosynthesis during embryonic induction and maturation include *PYRABACTIN RESISTANCE 1* (*PYR/PYL*), *2C protein phosphatase* (*PP2C*), *subfamily 2 Snfl-related kinases* (*SnRK2*) and *ABF*. *PYR/PYL*, encoding ABA receptors involved in ABA signal transduction [57], showed involvement throughout SE and exhibited significant upregulation. A key component of ABA biosynthesis is PP2C, which interacts with ABA receptors and SnRK2s [58]. In addition, biosyntheses of ET, brassinosteroid, jasmonic acid and salicylic acid are involved in SE in M. sativa, consistent with previous reports. Our transcriptome analysis revealed differential expression patterns of genes involved in hormonal signal transduction in SE in *M. sativa*. All hormonal signal transduction pathways are shown in Figure S3.

### 3.3. miRNA and Target Genes Form a Potential Molecular Regulatory Network in SE in M. sativa

**Figure 11:**
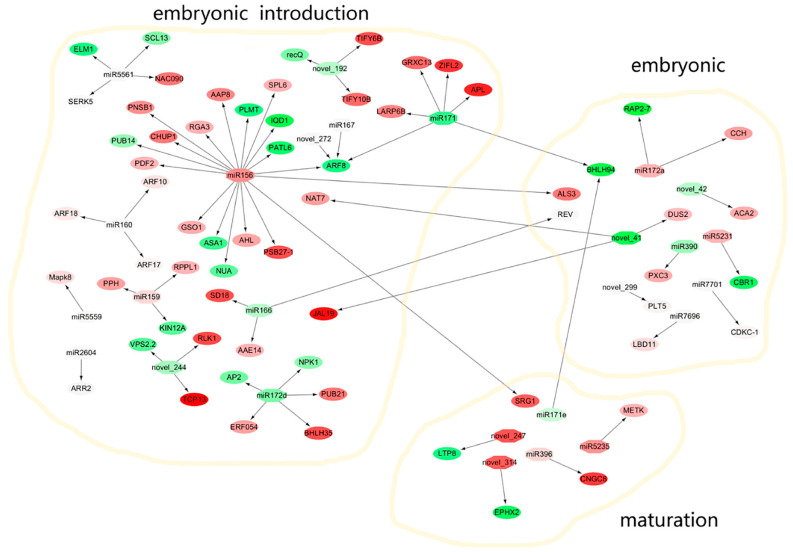
The miRNAs and target genes formed potential miRNA-target gene interaction network during embryonic induction, embyonic and maturation in *M.sative* SE. Red represents gene upregulation and green represents gene down regulation. The darker the color represent more obvious the difference in expression. Arrows represent the targeting of miRNAs.

The T0 generation cut cotyledon functions as an explant to induce somatic cell, and the addition of plant growth regulators to the medium promotes the formation of embryonic cells. *M. sativa* SE was divided into the embryonic induction, embryonic and maturation phases. Four types of calli (ICE, NEC, EC and CE) were selected to construct four transcriptome and small RNA libraries. The DEGs and differentially expressed miRNAs among four types of calli were identified. The expression levels of eight DEGs and eight miRNAs were detected by qRT-PCR. The target genes of the miRNAs were predicted and their functions annotated. The DEGs involved in the pathways of SE in *M. sativa* were predicted using KEGG analysis (Table S3). The miRNAs and target genes in the differentially expressed RNA libraries were compared. Previous reports suggested the existence of a potential molecular regulatory network, and the present study identified several novel genes and miRNAs involved in *M. sativa* SE (Figure 11).

The miR172 family is involved in a variety of processes including flowering time and floral organ identity [12], developmental timing [59], promotion of vegetative phase changes [60], soybean nodulation [61] and regulation of stem cells [62]. In addition, miR172 regulates *AP2* to control embryogenic and non-embryogenic callus development [63]. The miR172 target genes include *TOE1*, *TOE2*, *SMZ*, *SNZ* and SPL10 [59,64]. In our study, *AP2*, *NPK1*, *PUB21*, *ERF054* and *BHLH35* genes were found to be targeted by miR172d in embryonic induction, which might promote embryonic callus formation.

The miR156 family is involved in multiple plant developmental processes via targeting of *SPL* genes, including regulation of flowering [65], plastochrone length and organ size [66] and anthocyanin biosynthesis [20]. miR156 also regulates shoot development by targeting the SPL3 gene [67]. In addition, miR156 plays roles in SE. The *SPL10* and *SPL11* genes were repressed by miR156, which affects the precocious accumulation of maturation-phase transcripts, in Arabidopsis eight-cell embryos [20]. miR156 is also involved in early SE in the yellow poplar [18] and the regulation of CE formation [27,47]. Our results showed significantly upregulated miR156 expression in embryonic induction (Figure 8a) and identified *SPL6*, *AHL*, *ALS3*, *ARF8* and *SRG1* as target genes of miR156. However, aside from *SPL6*, the functions of the other target genes remain unclear.

miR159 regulates *LaMYB33* in the embryogenic formation and maturation stages of *larix kaempferi* SE [18]. In Arabidopsis, miR159 regulates GAMYB-like genes to promote programmed cell death [47]. In the present study, miR159 targeted *Pheophytinase* (*PPH*) in embryonic induction in *M. sativa*, suggesting that miR159 plays important roles in early SE.

In Arabidopsis, miR166 targeted *HD-ZIP III* TFs to regulate shoot apical meristem development during embryogenesis [68,69]. Moreover, miR166 regulated floral [70] and root development [71]. In *M. sativa* SE, miR166 targeted *AAE14* and *REV* in embryonic induction and maturation. In this study, miR166 was downregulated in embryonic induction; however, AAE14 was upregulated. Therefore, the miR166–AAE14 interaction may promote the transition from embryonic induction to embryonic formation. miR171 maintained embryogenic potential in *Larix kaempferi* Carr SE via regulation of its target gene *SCL6* [70]. In addition, miR171 targeted SCL6/22/27 to negatively regulate chlorophyll biosynthesis in Arabidopsis [72], and miR171 targeting of *GRAS* regulated GA and auxin homeostasis in the tomato [73]. In our study, *ARF8* was identified as a target gene of miR171 during embryonic induction. However, our results revealed that miR171 was upregulated, whereas *ARF8* was downregulated. *ARF8* is involved in auxin biosynthesis, which plays a crucial role in SE.

In addition, miR5561, miR390, miR5231, miR5559, novel_41 and novel_247 were differentially expressed during various stages of M. sativa SE (Table S10, Figure 11). According to predictions, miR5561 targeted *SERK5*, *Mitochondrial fission protein* (*ELM1*) and *NAC domain-containing protein 90* (*NAC090*) in embryonic introduction (Figure 11). miR390 was found to target ARF to regulate lateral root growth [74]. In our study, miR390 targeted *Leucine-rich repeat receptor-like tyrosine-protein kinase* (*PXC3*) in embryonic. Although we identified several novel miRNAs and target genes, further studies are needed to define their functions.

## 4. Materials and Methods

### 4.1. Plant Materials

SE consists of three major phases in *M. sativa*: embryonic induction (ICE to NEC), embryonic formation (NEC to EC) and maturation (EC to CE). EC was induced in MS medium supplemented with 0.2 mg/L 6-benzylaminopurine and 4 mg/L 2,4-D. Then, the embryos were collected at 0, 10 and 40 d, representing the ICE, NEC and EC stages, respectively. CEs were obtained in MS medium containing 1 mg/L kinetin and 0.5 mg/L 6-benzylaminopurine and collected at 69 d. Calli were induced from cotyledons as explants. All samples included three biological replicates.

### 4.2. Morphological Analysis

We compared tissue morphology at different periods of SE using a light microscope. An explant showing significant changes was selected for further study. The organization of different morphological features was observed using a stereomicroscope (MZ FLIII; Leica, Wetzlar, Germany). The sample explants at different phases (0, 3, 6, 12, 24, 48 and 72 h, and 5, 7, 10, 15, 25, 40 and 69 d) were fixed in glutaraldehyde. Samples were stained with safranin for 1–2 h and rinsed with water. Then, a 50%, 70% and 80% alcohol gradient was applied for 1 min to decolorize the samples. Paraffin sections of the samples were made for observation under a light microscope (ECLIPSE Ci-L, NIKON, Tokyo, Japan).

### 4.3. RNA Extraction

Total RNA was extracted from the explants during the ICE, NEC, EC and CE phases using TRIzol reagent (Invitrogen, Burlington, ON, Canada) according to the manufacturer’s protocol. RNA quality was visualized on a 1% agarose gel. RNA purity was measured using a NanoPhotometer spectrophotometer (IMPLEN, Westlake Village, CA, USA). RNA concentrations were measured using the Qubit RNA Assay Kit in a Qubit 2.0 Fluorometer (Life Technologies, Carlsbad, CA, USA). RNA integrity was assessed using the Bioanalyzer 2100 system (Agilent Technologies, Santa Clara, CA, USA).

### 4.4. Sample Preparation for Sequencing and Data Quality Control

RNA (6 μg per sample) was used as input material for the RNA sample preparations. All four samples had RNA integrity number (RIN) values above 8.0. Transcriptome and small RNA libraries of ICE, NEC, EC and CE were constructed and sequenced using the Illumina system [75,76]. Raw data (raw reads) in fastq format were processed using in-house Perl scripts. In this step, we obtained the clean data (clean reads), and removed reads containing the adapter, reads containing poly-N, and low-quality reads from the raw data. Simultaneously, Q20, Q30, GC content, and the sequence duplication level of the clean data were calculated. All the subsequent analyses were based on clean data.

### 4.5. Annotation of DEGs and miRNAs

To obtain DEGs from ICE to NEC, NEC to EC and EC to CE, the fold change (FC) in expression was assessed by taking the log2 ratio of Reads Per Kilobase per Million mapped reads (RPKM). Differential expression analysis of two conditions was performed using the DEGSeq R package (1.12.0; TNLIST, Beijing, China). The adjustment of *p*-values was performed using the Liszkay method [77]. The corrected *p*-value of 0.005 and log2 of ±1 were set as the threshold for significant differential gene expression. DEGs were annotated using Blast2GO v2.5 (BioBam, Valencia, Spain). The genes and miRNA expression patterns of each phase (ICE, NEC, EC and CE) were clustered according to their log2 value using corset v1.05 software (Melbourne, Australia), and the database used for comparison was union_for_cluster. Heat-maps of cluster data were constructed using Java Tree View (Stanford, CA, USA) [78]. miRNAs were annotated using the Rfam database, and novel miRNAs were predicted using miREvo and miRdeep2 software. miRNA target genes were predicted using the psRNATarget server. The miRNA–target interaction network was drawn using cityscape (San Diego, CA, USA).

### 4.6. Functional Annotation of DEGs and miRNAs

GO analysis was performed using GOseq [79]. and the GO database (http://www.geneontology.org/, accessed on 5 June 2022). The KOG/COG database can be found at (http://www.ncbi.nlm.nih.gov/COG/, accessed on 5 June 2022), and diamond v0.8.22 was used as the analysis software for the KOG database. KEGG classification was constructed based on the KEGG database (http://www.genome.jp/kegg/, accessed on 5 June 2022). The KOBAS (Beijing, China) was used for the KEGG analysis.

### 4.7. Indirect Competitive Enzyme-Linked Immunoassay (icELISA) for Detection of Indole-3-Acetic Acid (IAA), Gibberellin (GA3), Zeatin Riboside (ZR) and Abscisic Acid (ABA)

The icELISA protocol was previously described [80]. Reagents purchased from Sigma-Aldrich (St. Louis, MO, USA) included IAA, GA3, ZR, ABA, goat anti-mouse IgG conjugated to horseradish peroxidase (IgG-HRP), o-phenylenediamine, potassium periodate and citric acid monohydrate (C_6_H_8_O_7_·H_2_O). SE was divided into four phases (ICE, NEC, EC and CE), and each group of *M. sativa* explants (8.0 g) was prepared for detection using icELISA. IAA, GA3, ZR and ABA were extracted as previously reported [81]. Detected samples were used for icELISA analysis.

### 4.8. Quantitative RT-PCR

Multiple genes and miRNAs were sorted for validation using reverse transcriptase-polymerase chain reaction (RT-PCR) (Bio-Rad C1000, Hercules, CA, USA). The genes and miRNA primers (Table S14) were designed using primer premier 5.0. The 18S RNA was used as an internal control for gene validation [82]. The small nuclear RNA (snRNA) U6 was used as an internal control for miRNA validation [83]. Quantitative RT-PCR (qRT-PCR) was performed as described by Liu et al. [84].

### 4.9. Statistical Analyses

Differences in the means for hormone detection were assessed by one-way Student’s *t*-test at the 0.05 significance level. Differences in the means for gene expression data were assessed by one-way ANOVA at the 0.05 significance level.

## 5. Conclusions

Analyses of small RNAs and transcriptomes involved in the somatic embryonic induction, embryonic and maturation phases of SE provided information regarding the molecular mechanisms specific to *M. sativa*. Morphological observations revealed significant changes in tissues in SE, particularly during the stages of ICE, NEC, EC and CE. Several novel DEGs were identified in *M. sativa* SE, including *LBD*, *AGO* and *AGL*, which play important roles in promoting embryonic formation. Our analysis suggested that many DEGs playing roles in plant hormone signal transduction are involved in regulatory processes, e.g., *IAA* and *ARF* regulation of auxin biosynthesis, *LEC2* regulation of GA biosynthesis, and *MYB4* and *MYB48* regulation of ABA biosynthesis. In addition, hormonal signal transduction is regulated by the interactions of target genes with various miRNAs such as miR156, miR160 and miR167. This study predicted 110 novel miRNA families involved in embryonic induction, embryonic and maturation. Further studies are needed to determine whether these novel miRNAs play additional roles in *M. sativa*. Finally, we analyzed several miRNAs exhibiting significantly different expression patterns in SE and predicted their target genes. A potential miRNA–target gene interaction network is presented in Figure 11, which outlines the molecular mechanisms of SE in *M. sativa*.

## Figures and Tables

**Table 1:** Transcriptome data quality profile.

Sample	Raw Reads	Clean Reads	Clean Bases	Error (%)	Q20 (%)	Q30 (%)	GC (%)
ICE_1	52,384,258	51,607,148	7.74G	0.01	97.75	94.00	41.28
ICE_2	58,306,722	56,696,602	8.5G	0.02	97.20	92.69	41.30
ICE_3	50,810,168	50,103,162	7.52G	0.01	97.86	94.21	41.34
NEC_1	57,200,884	55,844,904	8.38G	0.01	97.89	94.30	41.60
NEC_2	60,219,472	56,035,448	8.41G	0.02	96.97	92.05	41.86
NEC_3	53,763,200	52,838,680	7.93G	0.01	97.97	94.50	41.60
EC_1	52,977,216	51,478,324	7.72G	0.01	97.97	94.51	41.45
EC_2	55,361,562	54,341,900	8.15G	0.01	98.02	94.63	41.39
EC_3	57,268,222	55,969,446	8.4G	0.01	97.64	93.76	41.79
CE_1	63,997,186	62,745,116	9.41G	0.02	97.47	93.47	41.93
CE_2	50,846,076	49,993,036	7.5G	0.02	97.49	93.49	41.95
CE_3	55,045,608	54,018,626	8.1G	0.02	97.44	93.39	41.65

ICE, non-embryogenic callus; NEC, non-embryogenic callus; EC embryogenic callus; CE, cotyledon embryo; Q20 , phred percentage of base greater than 20, pecentage of base population; Q30, phred percentage of base greater than 30, percentage of base population; GC, GC content.

**Table 2:** Small RNA filter profile.

Sample	ICE	NEC	EC	CE
total reads	14,669,757 (100.00%)	14,696,331 (100.00%)	11,817,247 (100.00%)	10,282,021 (100.00%)
N% > 10%	18 (0.00%)	25 (0.00%)	0 (0.00%)	0 (0.00%)
low quality	753,071 (5.13%)	1,954,044 (13.30%)	488,075 (4.13%)	225,087 (2.19%)
5 adapter contamine	23,386 (0.16%)	16,804 (0.11%)	9703 (0.08%)	11,016 (0.11%)
3 adapter null or insert null	649,842 (4.43%)	316,352 (2.15%)	212,613 (1.80%)	189,171 (1.84%)
With ployA/T/G/C	39,873 (0.27%)	55,440 (0.38%)	35,208 (0.30%)	32,043 (0.31%)
clean reads	13,203,567 (90.01%)	12,353,666 (84.06%)	11,071,648 (93.69%)	9,824,704 (95.55%)

**Table 3:** Annotation of small RNA.

Types	ICE	NEC	EC	CE
known miRNA	740,026 (12.40%)	302,053 (5.92%)	299,136 (6.74%)	479,121 (11.25%)
novel miRNA	132,783 (2.23%)	63,563 (1.25%)	34,189 (0.77%)	46,934 (1.10%)
rRNA	432,250 (7.24%)	121,355 (2.38%)	252,580 (5.69%)	149,802 (3.52%)
tRNA	3 (0.00%)	0	1 (0.00%)	0
snRNA	3988 (0.07%)	2290 (0.04%)	5984 (0.13%)	2902 (0.07%)
snoRNA	9607 (0.16%)	23,276 (0.46%)	22,688 (0.51%)	15,722 (0.37%)
TAS	19,322 (0.32%)	31,369 (0.61%)	25,992 (0.59%)	132,783 (0.46%)
other	4,629,365 (77.58%)	4,560,608 (89.34%)	25,992 (0.59%)	19,645 (0.46%)
total	5,967,344	5,104,514	4,436,671	4,257,086

## Data Availability

The raw assembled data has been uploaded The National Center for Biotechnology Information (NCBI). The accession number is PRJNA474427. The URL is https://www.ncbi.nlm.nih.gov/bioproject/474427, accessed on 5 June 2022.

## Supplementary Materials

The following supporting information can be downloaded at: https://www.mdpi.com/article/10.3390/ijms23158633/s1.
