# Supplementary material for: Uncovering a Phenomenon of Active Hormone Transcriptional Regulation during Early Somatic Embryogenesis in Medicago sativa
Source: Int J Mol Sci. 2022 Aug 3;23(15):8633. doi: 10.3390/ijms23158633 (PMC9368939; doi:10.3390/ijms23158633)
Supplement: Supplementary file 1 [file ijms-23-08633-s001.zip › Supplementary Material/Figure S2 KOG function category in Medicago sativa.pdf]

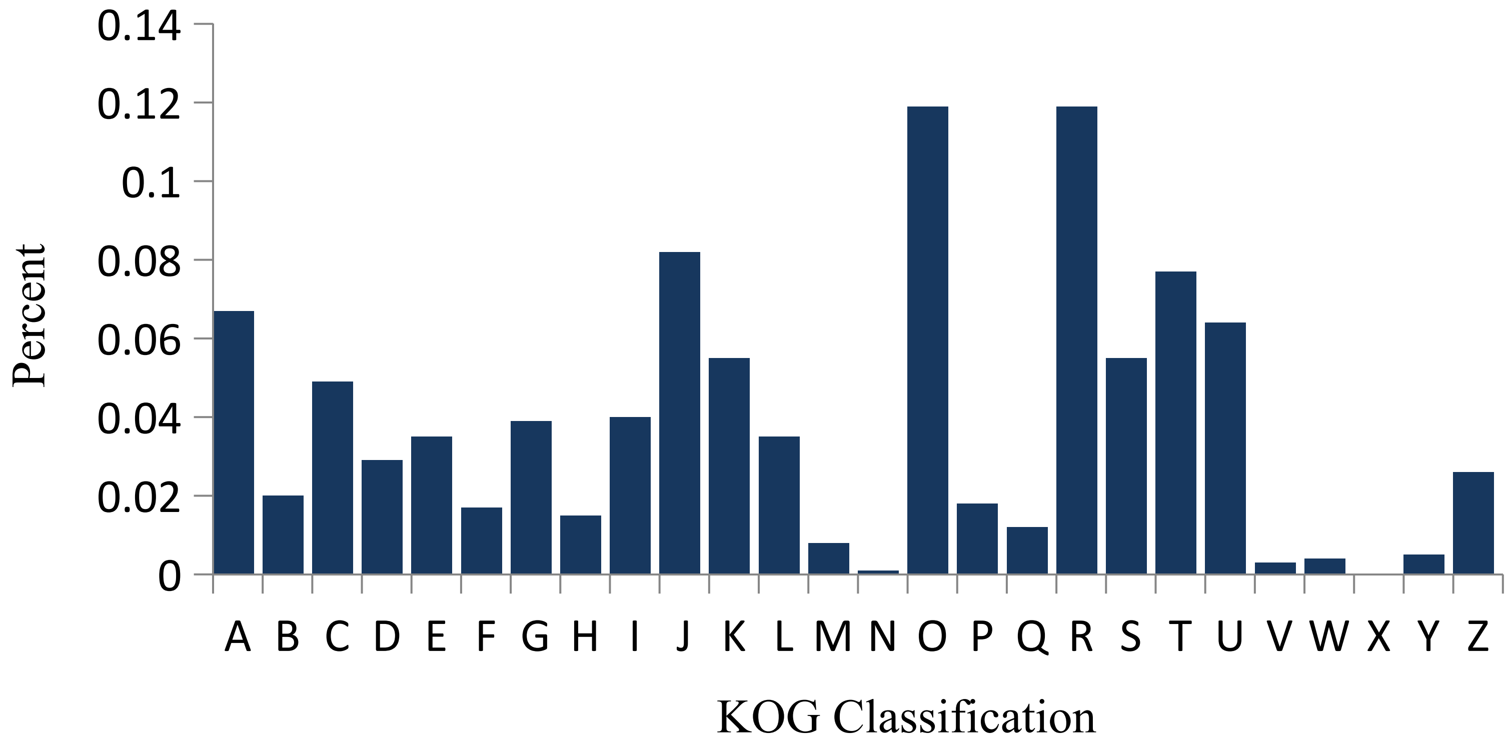

Figure S2. KOG function category in *Medicago sativa*.

A: RNA processing and modification; B: Chromatin structure and dynamics; C: Energy production and conversion; D: Cell cycle control, cell division, chromosome partitioning; E: Amino acid transport and metabolism; F: Nucleotide transport and metabolism; G: Carbohydrate transport and metabolism; H: Coenzyme transport and metabolism; I: Lipid transport and metabolism; J: Translation, ribosomal structure and biogenesis; K: Transcription; L: Replication, recombination and repair; M: Cell wall/membrane/envelope biogenesis; N: Cell motility; O: Posttranslational modification, protein turnover, chaperones; P: Inorganic ion transport and metabolism; Q: Secondary metabolites biosynthesis, transport and catabolism; R: General function prediction only; S: Function unknown; T: Signal transduction mechanisms; U: Intracellular trafficking, secretion, and vesicular transport; V: Defense mechanisms; W: Extracellular structures; X: Unamed protein; Y: Nuclear structure; Z: Cytoskeleton.
