# Supplementary figures and images for: Uncovering a Phenomenon of Active Hormone Transcriptional Regulation during Early Somatic Embryogenesis in Medicago sativa
Source: Int J Mol Sci. 2022 Aug 3;23(15):8633. doi: 10.3390/ijms23158633 (PMC9368939; doi:10.3390/ijms23158633)

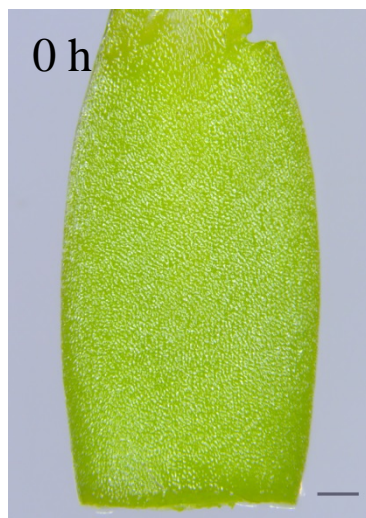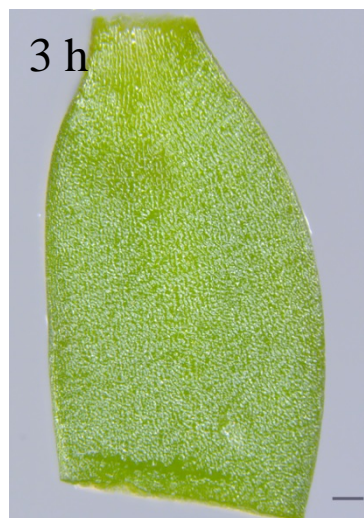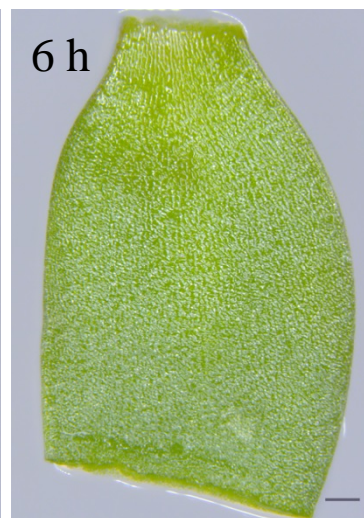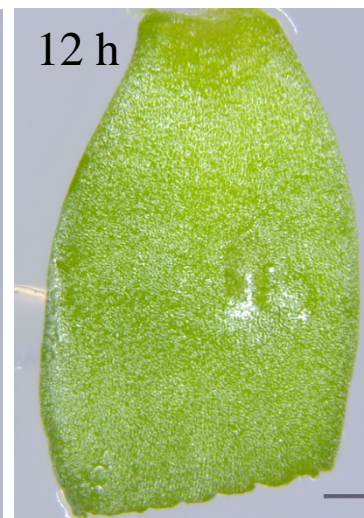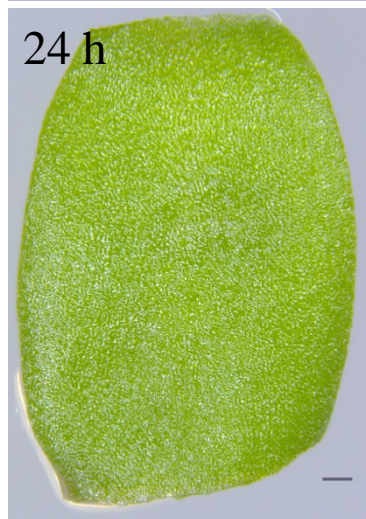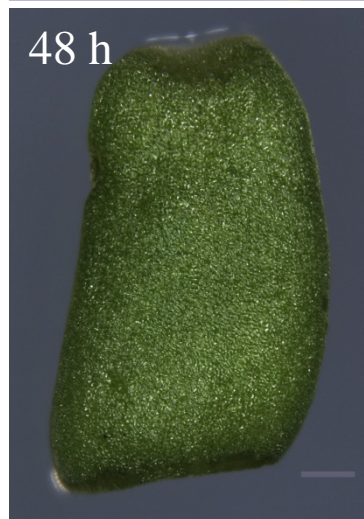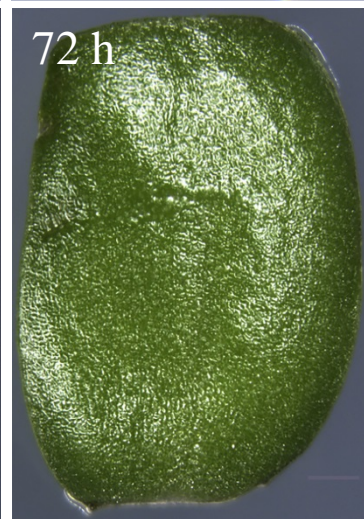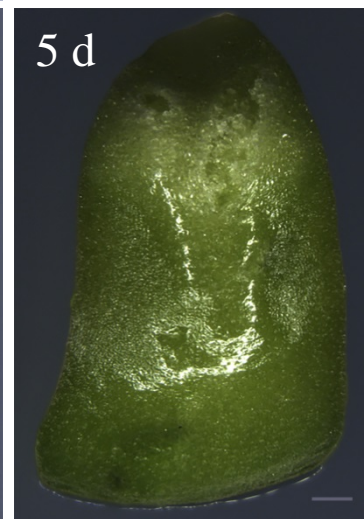

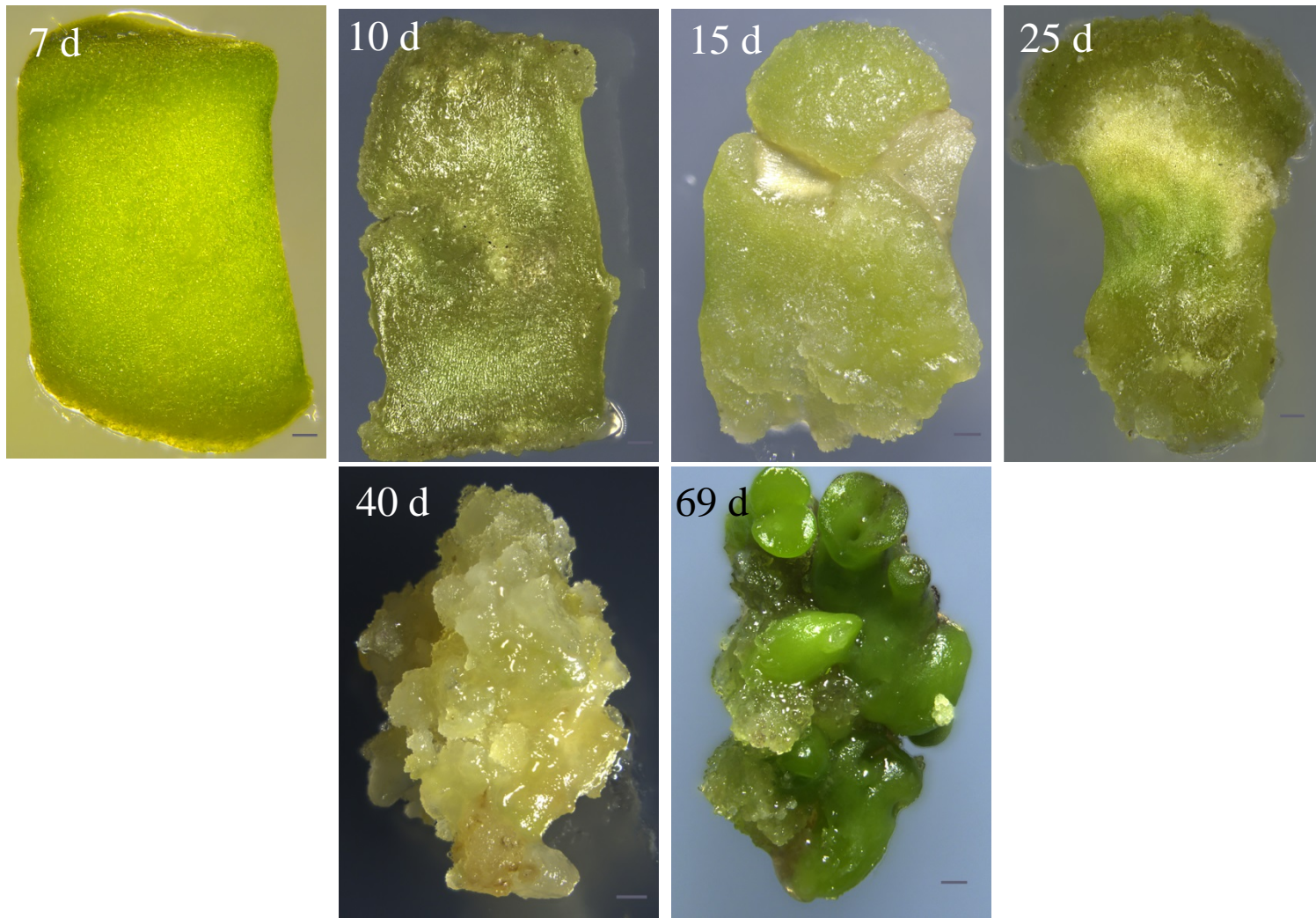

**Figure S1.** the change of morphology in *Medicago sativa* SE. They included 0h, 3h, 6h, 12h, 24h, 48h, 72h, 5d, 7d, 10d, 15d, 25d, 40d and 69d.

Supplement: Supplementary file 1 [file ijms-23-08633-s001.zip › Supplementary Material/Figure S1 the change of morphology in Medicago sativa SE..pdf]

# PLANT HORMONE SIGNAL TRANSDUCTION

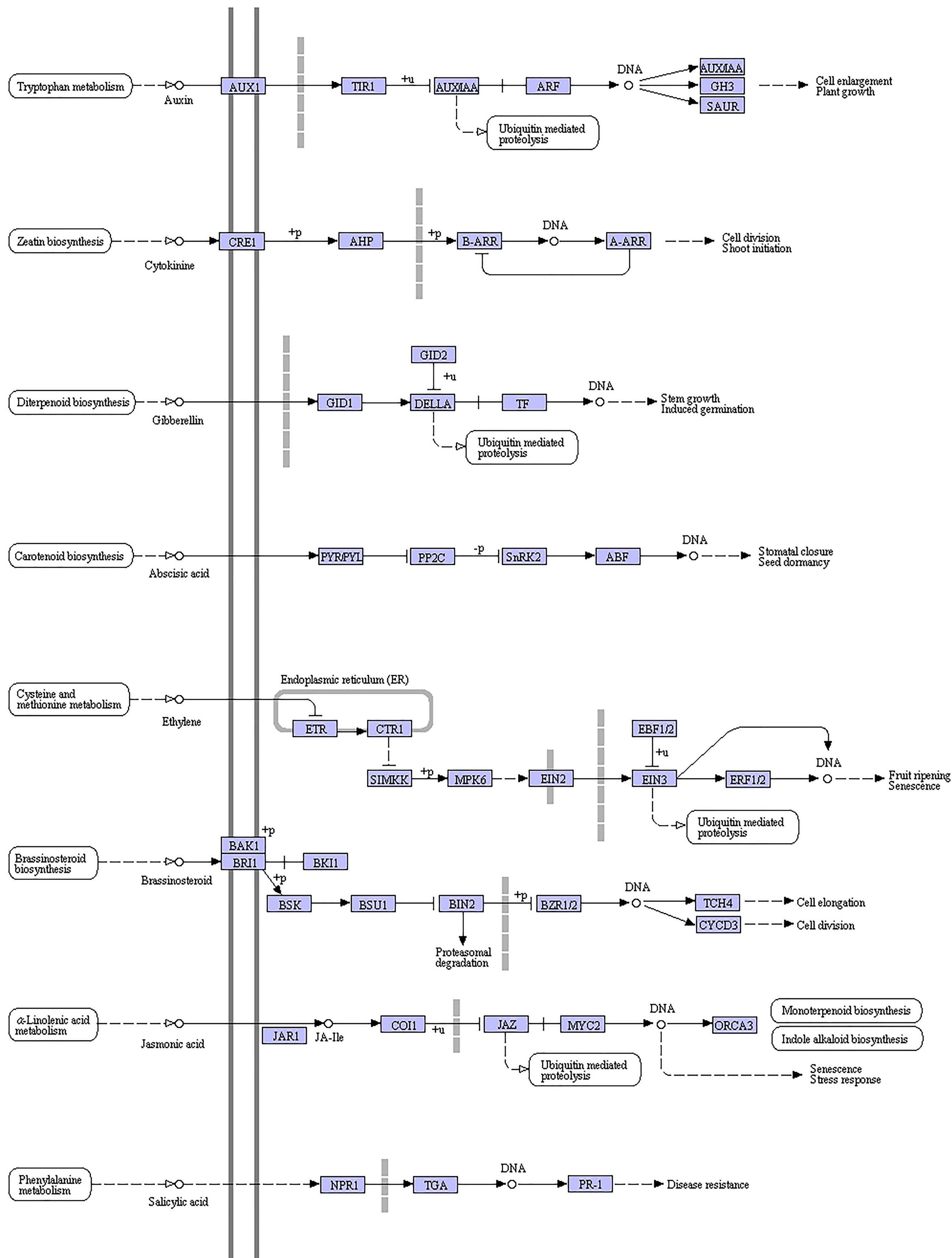

Supplement: Supplementary file 1 [file ijms-23-08633-s001.zip › Supplementary Material/Figure S3 Plant hormone signal transduction pathway.pdf]
